# Differences in Imaging and Histology Between Sinonasal Inverted Papilloma with and Without Squamous Cell Carcinoma

**DOI:** 10.3390/diagnostics15131645

**Published:** 2025-06-27

**Authors:** Niina Kuusisto, Jaana Hagström, Goran Kurdo, Aaro Haapaniemi, Antti Markkola, Antti Mäkitie, Markus Lilja

**Affiliations:** 1 Department of Oral Pathology and Radiology, University of Turku, FI-20520 Turku, Finland; jaana.hagstrom@utu.fi; 2 Department of Pathology, Helsinki University Hospital, FI-00029 Helsinki, Finland; 3 Department of Medical Imaging Center, Helsinki University Hospital, FI-00029 Helsinki, Finland; goran.kurdo@hus.fi (G.K.); antti.markkola@hus.fi (A.M.); 4 Department of Otorhinolaryngology—Head and Neck Surgery, University of Helsinki and Helsinki University Hospital, FI-00029 Helsinki, Finland; aaro.haapaniemi@hus.fi (A.H.); antti.makitie@hus.fi (A.M.); markus.lilja@hus.fi (M.L.); 5 Research Program in Systems Oncology, Faculty of Medicine, University of Helsinki, FI-00014 Helsinki, Finland

**Keywords:** sinonasal, inverted papilloma, squamous cell carcinoma, computed tomography

## Abstract

**Objectives:** Sinonasal inverted papilloma (SNIP) is a rare benign tumor that has potential for malignant transformation, usually into squamous cell carcinoma (SCC). The pre-operative differentiation between SNIP and SNIP-SCC is essential in determining the therapeutic strategy, but it is a challenge, as biopsies may fail to recognize the malignant part of the tumor. Further, a SNIP can also be locally aggressive and thus mimic a malignant tumor. This retrospective study compares the pre-operative differences in computed tomography (CT) and histologic findings between patients with a benign SNIP and those with a SNIP-SCC. **Methods:** Eight patients with SNIP-SCC were selected from the hospital registries of the Department of Otorhinolaryngology, Helsinki University Hospital (Helsinki, Finland). For each case a comparable SNIP case without malignancy was selected. Five histopathologic samples of both the SNIP and SNIP-SCC tumors were retrieved. CT images and the histopathologic samples were re-evaluated by two observers. **Results:** The nasal cavity and ethmoid and maxillary sinuses were the most common sites for both tumor types. The SNIP tumors were mostly unilateral, and the SNIP-SCC tumors were both unilateral and bilateral. Only SNIP-SCC tumors demonstrated bone defects and orbital or intracranial invasion. Dysplastic findings such as dyskeratosis, nuclear atypia, and maturation disturbances were seen only in the SNIP-SCC tumors. **Conclusions:** Bony destruction and invasion of adjacent structures in pre-operative CT images seem to be pathognomonic signs of SNIP-SCC based on this series. To differentiate between SNIP and SNIP-SCC tumors all available pre-operative investigations are warranted.

## 1. Introduction

Sinonasal inverted papilloma (SNIP) is a rare benign tumor that constitutes between 0.4% and 7% of all sinonasal tumors [1]. Men are more commonly affected than women, and SNIP commonly occurs in the fifth to sixth decades of life [2,3].

SNIP has a recurrence rate of 2–15% [4,5,6] and 7–10% risk of malignant transformation, mainly into squamous cell carcinoma (SCC) [7,8]. SCC can be associated with SNIP synchronously (7.1%) or metachronously (3.6%) [8]. There may be geographical variation in the incidence of SNIP-associated malignancy, but reliable research on this is scarce. The incidence of SNIP-associated malignancy according to Nakamura et al. is significantly higher in East and Southeast Asia (11.0%) and North America (10.4%) compared to Europe (3.9%) [9].

The etiology of SNIP is unclear, but potential risk factors, especially human papillomavirus (HPV), are widely studied [10]. Also, smoking, environmental and industrial exposures, and chronic inflammation are suspected to have a role in its etiology [10].

Pre-operative diagnosis between SNIP and SNIP-associated SCC (SNIP-SCC) is a challenge but essential in determining the therapeutic strategy. A comprehensive pre-operative clinical assessment, including nasal endoscopy and tumor biopsy, remain crucial. However, SCC may not involve the entire tumor, and pre-operative biopsies may not detect the malignant part of the tumor. Hence, pre-operative radiographic imaging plays an important role in sinonasal tumor diagnosis, and computed tomography (CT) is the preferred initial imaging method. The benefits of CT imaging include its speed, wide availability, and its crucial role in examining bone structures. Bony erosion on CT images indicates aggressive tumor growth [11]. However, SNIP can be locally aggressive, causing bony destruction, and it is difficult to differentiate between SNIP and SNIP-SCC tumors on CT alone [12]. Magnetic resonance imaging (MRI) has shown promise for aiding in differential diagnosis [13,14]. Generally, MRI acts as a complementary imaging method to CT, as it is better in differentiating soft tissues. Despite this, MRI is not always available pre-operatively because of poor accessibility at some institutions. Additionally, MRI safety concerns may limit the use of MRI scans for certain patients [15].

This retrospective study aims to compare the characteristic pre-operative differences in CT and histological findings between patients with benign SNIP and those with SNIP-SCC. Furthermore, the study discusses the latest imaging methods and their applications in distinguishing SNIP-SCC pre-operatively.

## 2. Materials and Methods

**Figure 1:**
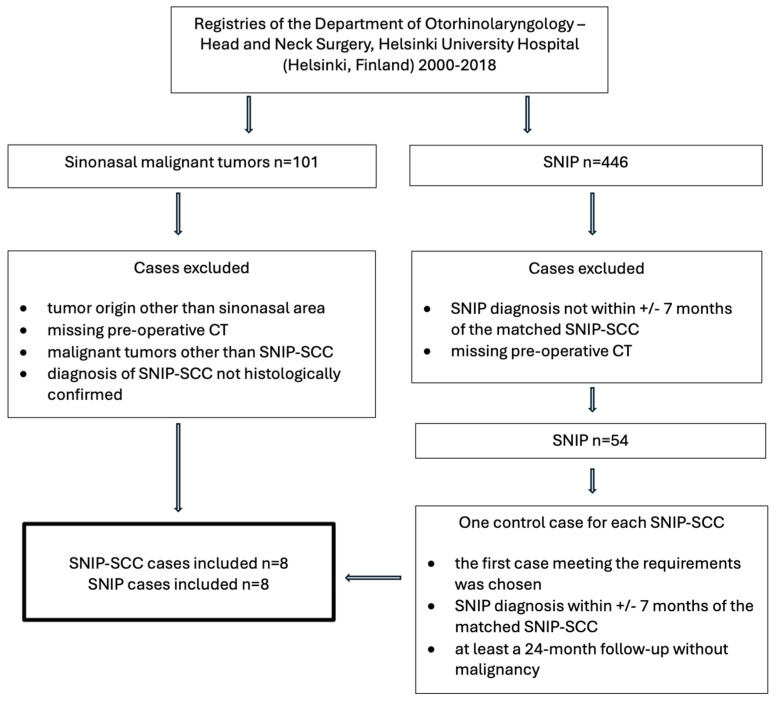
Selection process of the SNIP and SNIP-SCC cases.

This was a single-institution retrospective study at the Hospital District of Helsinki and Uusimaa (HUS, Helsinki, Finland) evaluating patients with SNIP-SCC and SNIP between 2000 and 2018 (research permission HUS/146/2020). SNIP-SCC and SNIP cases were manually selected from the hospital registries of the Department of Otorhinolaryngology—Head and Neck Surgery, Helsinki University Hospital (Helsinki, Finland). Inclusion criteria were as follows: histologically confirmed SNIP or a malignant SNIP-SCC associated with SNIP tumor. Cases involving tumors other than SNIP and malignancies other than SCC were excluded. Additionally, a pre-operative CT image was required for all cases. We identified eight patients with SNIP-SCC and selected a matched case of SNIP without malignancy for each SNIP-SCC case. The benign SNIP cases were required to have a 24-month follow-up without malignancy, and the SNIP diagnosis had to be made within +/−7 months of the matched SNIP-SCC. Figure 1 illustrates the case selection process. At the selection stage of the cases, tumor location or size were not yet considered. We compared data from the eight patients with SNIP-SCC and the eight patients with benign SNIP. Data included age at diagnosis, sex, tumor site, recurrence, and follow-up duration in months. We gathered the dates of SNIP and SCC diagnosis to investigate whether the SCC was synchronous or metachronous (Table 1). Information regarding smoking history, sinusitis, symptoms, surgical treatment, radiation therapy, or chemotherapy were not collected.

Preoperative sinonasal CT images were reviewed by two authors, a senior radiologist, G.K., and an oral and maxillofacial radiologist, N.K. Tumor location, size, and shape were analyzed from CT images as well as bone defects, calcification, and hyperostosis (Table 2). All scans were reviewed on a tri-planar reconstruction—axial, sagittal, and coronal. Twelve out of the 16 cases had been performed with contrast imaging. This study did not review pre-operative MRIs, as they were available only in 5/8 of the SNIP-SCC cases and 3/8 of the SNIP cases.

Initially, a pathologist had examined all the cases, and the selection of cases was based on the pathology report. However, not all histological samples could be retrieved for re-evaluation. Five samples of both the SNIP and SNIP-SCC tumors were retrieved from the Helsinki Biobank archives (research permission HBP20210141). The histopathologic samples were re-evaluated by a senior oral pathologist, J.H., and the first author, N.K. The diagnosis of SNIP was ensured from all the samples, and the histopathologic findings were analyzed in detail, as shown in Table 3.

## 3. Results

Six of the eight patients with SNIP-SCC tumors were synchronous SCC, and two were metachronous. Detailed information on clinical characteristics is shown in Table 1.

**Figure 2:**
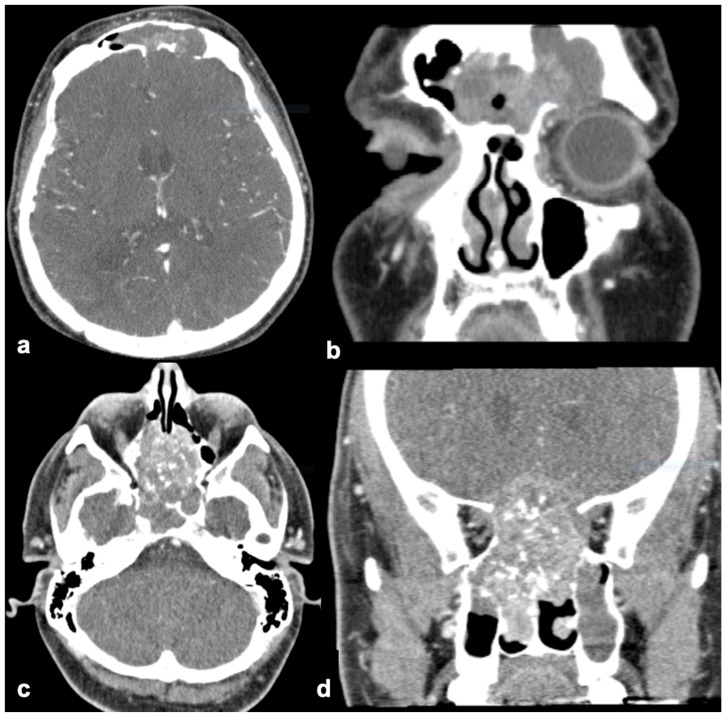
(**a**–**d**) Axial (left) and coronal (right) slices of two CT images of SNIP-SCC (case 1 (**a**,**b**) and case 2 (**c**,**d**)). Intracranial defects are observed in (**a**) posterior wall of frontal sinus and (**b**) skull base. Both tumors are bilateral (extending to both sides).

**Figure 3:**
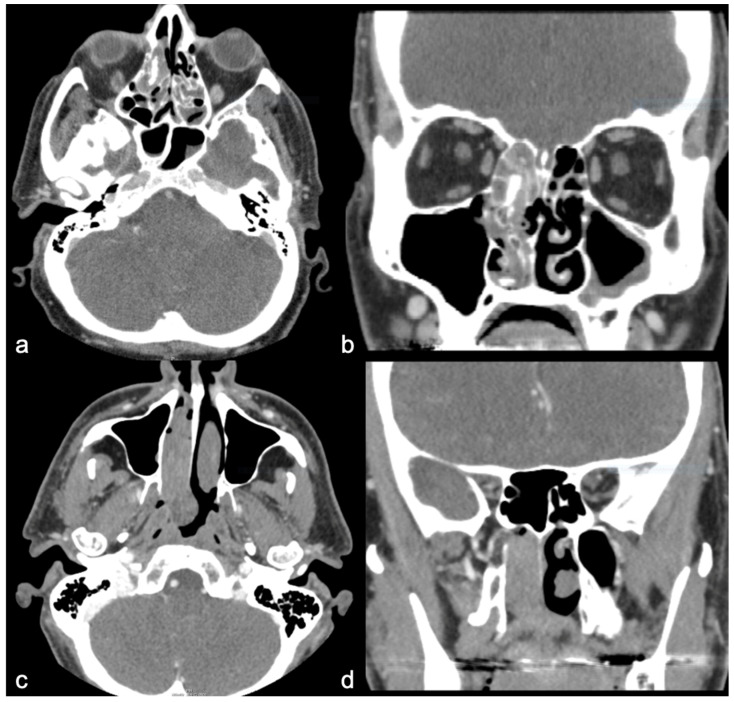
(**a**–**d**) Axial (left) and coronal (right) slices of two CT images of SNIP without SCC (case 1 (**a**,**b**) and case 2 (**c**,**d**)). Both tumors are unilateral (affecting only one side) without clear bone defects.

The nasal cavity and ethmoid and maxillary sinuses were the most common sites for both tumor types. The SNIP tumors were mostly unilateral (7/8), and the SNIP-SCC tumors were both unilateral (4/8) and bilateral (4/8) (Figure 2 and Figure 3). Orbital or intracranial invasion was demonstrated only in SNIP-SCC tumors (2/8), one with intracranial invasion and one with both orbit and intracranial invasion (Figure 2). Only SNIP-SCC tumors demonstrated bone defects (6/8). The shape of the tumor was more often polypoid in the SNIP tumors (6/8) than in the SNIP-SCC (4/8). The surface of the SNIP and SNIP-SCC tumors was mainly rough. The mean diameter of the SNIP tumors was 48 mm, and it was 57 mm for the SNIP-SCC tumors. Calcification was equally seen in both tumors, but hyperostosis was seen more often in the SNIP tumors (4/8) than in the SNIP-SCC tumors (2/8). In 2/8 of the SNIP cases, the hyperostosis site on CT was found to be the site of tumor attachment at the surgery. In 1/8 of the SNIP-SCC cases, the hyperostosis site in CT corresponded with the attachment site at the surgery. A contrast-enhanced examination was performed in 7/8 of the SNIP-SCC cases and in 5/8 of the SNIP cases, and the CT enhancement was seen in both SNIP and SNIP-SCC tumors. Detailed information on CT findings is shown in Table 2.

A total of 10 histologic samples were examined—five cases from both groups. Dysplastic findings such as dyskeratosis, nuclear atypia, and maturation disturbances were seen only in the SNIP-SCC tumors. Additionally, basaloid hyperplasia, mitosis in the basal cell layer, loss of epithelial stratification, mitosis above the basal cell layer, and goblet cells were seen more in the SNIP-SCC tumors. The number of neutrophils, clear cell cytoplasm, respiratory epithelium, and squamous cell metaplasia was higher in the SNIP tumors. We found no atypical keratinization, apoptotic keratinocytes, or atypical mitoses in either group. Detailed information is presented in Table 3.

### Statistical Analysis

Because of the small number of cases, the statistical analysis remained inconclusive.

## 4. Discussion

We investigated the imaging characteristics that would aid in differentiating between sinonasal inverted papilloma (SNIP) and SNIP-associated squamous cell carcinoma (SNIP-SCC). The findings in CT images that were only found in SNIP-SCC tumors were orbital and intracranial invasion and bone defects typically located in the nasal and maxillary walls (Figure 2). The nasal wall and ethmoid and maxillary sinuses were the most common tumor location sites for both tumors, but the SNIP tumors were mostly unilateral, and the SNIP-SCC tumors were both unilateral (4/8) and bilateral (4/8) (Figure 1 and Figure 2). Our findings from the pre-operative CT images of SNIP-SCC align with the recent studies of Park and coworkers, which indicate that bony destruction, remodeling, and invasion of adjacent structures may be the signs of SNIP-SCC [16].

In the histologic samples, dysplastic findings such as dyskeratosis, nuclear atypia, and maturation disturbances were seen only in the SNIP-SCC tumors. The clinical characteristics of this study are in accordance with previous studies [4,6,7]. The mean follow-up time was longer with SNIP tumors than with SNIP-SCC tumors. This may result from the tendency observed in the group of SNIP tumors to present recurrences.

CT is essential in investigating bone structures, and it has long been preferred as the primary imaging method because of its large availability. The radiographic findings of SNIP are a unilateral lobulated mass within the nasal cavity near the middle meatus, extending into the maxillary sinus [6,17]. SNIP is characterized by centrifugal growth, and larger tumors demonstrate bone remodeling or local bony destruction [12]. Localized hyperostosis in CT images may reflect the tumor’s origin or entrapped bone, or tumor calcification may occur [18]. SNIP shows mild heterogenous enhancement in CT [17,18]. Azuma et al. (2019) studied the relative CT numbers for differentiating SCC and SNIP tumors [19]. They found that the relative CT numbers are higher in SCC than in SNIP tumors, which could give a diagnostic clue for the differentiation of SNIP and SCC tumors [19]. However, CT alone has limitations in distinguishing SNIP from SNIP-SCC and the tumor from inflammatory conditions [20,21]. Bone erosion or destruction detected in CT can also be present with benign SNIP tumors and cannot reliably predict malignancy [11,12,18]. Our study observed bone defects only in SNIP-SCC cases, and similar findings were observed in the study of Umeda et al. [22]. They evolved a CT scoring system revealing that bone destruction and erosion could differentiate SNIP-SCC from IP. Scoring systems will be an important practical tool alongside deep learning, especially with patients who cannot undergo MRI scanning.

MRI serves as a complementary imaging method along with CT, as it can separate soft tissue from secretions [23,24]. In MRI T1-weighted images, SNIP appears iso- to hypointense, and in T2-weighted images, hyperintense to muscles [18]. A convoluted cerebriform pattern (CCP) of enhancement is a typical feature of SNIP in MRI [14,18]. However, this can also be seen in malignant tumors, and it is not a reliable sign for benign SNIP [20]. MRI can demonstrate the intracranial extension of the tumor, as well as necrosis and secondary inflammatory signs [18,25]. According to Ojiri and coworkers, necrosis observed in imaging strongly suggests for SCC [26]. Apparent diffusion coefficient (ADC) values seem promising in differentiating between SNIP and SNIP-SCC tumors pre-operatively [13,14]. Dynamic contrast-enhanced (DCE) MRI can be helpful for distinguishing benign and malignant tumors, as malignant ones exhibit a washout-type time intensity curve (TIC) [27]. Although MRI is a promising pre-operative imaging method for recognizing the malignant transformation of SNIP [20], several studies still recommend CT and MRI imaging for distinguishing between benign and malignant SNIP [14,18,28]. MRI images were not included in this study, because they were available only in a few cases. Detailed pre-imaging safety protocols and locally limited access to pre-operative MRI imaging may explain the number of MRI images. MRI safety risks, such as projectile injury, an excessive specific absorption rate (SAR), and thermal burns, can be prevented by proper pre-imaging screening and the training of personnel [15].

In the future, positron emission tomography (PET) CT might be helpful pre-operatively. FDG uptake has been found to be higher in malignant tumors than in benign tumors [29]. However, FDG uptake is also high in benign sinonasal papillomas, especially in oncocytic papillomas [29]. Currently, PET-CT cannot reliably distinguish between benign SNIP and SNIP-SCC, but PET-CT imaging methods are continuously developing and might be helpful in future.

Histopathology is still the golden standard required for the definite diagnosis of SNIP and its malignant transformation [20,30]. The histopathologic features of SNIP consist of squamous epithelial proliferation into the submucosal stroma with inverting islands of epithelium. The stroma of fibrous connective tissue can include inflammatory cells, and the epithelium can include goblet cells and mucin-filled microcysts [7]. The malignancy of SNIP tumors is shown to be related to the increase in hyperkeratosis, the presence of squamous epithelial hyperplasia, an increase in the mitotic index, and a decrease in eosinophils [31]. Specific histopathologic parameters of SNIP have not been found to reliably predict recurrence or malignant transformation, and therefore, a long-term follow-up is necessary [30]. In our study, the number of neutrophils, clear cell cytoplasm, respiratory epithelium, and squamous cell metaplasia were greater in the SNIP tumors. Dysplastic findings such as dyskeratosis, nuclear atypia, and maturation disturbances were seen only in the SNIP-SCC tumors.

This study was conducted at a single institution, which is a significant limitation due to the limited number of SNIP-SCC cases and the data involved. The missing imaging data and histopathologic samples may have been processed at other healthcare organizations. MRI images were not included, because they were only available for a few cases. Symptoms, clinical evaluations, or treatment protocols were not included in this study. It is also notable that the timeline of this retrospective study is broad, which may have an influence on the recorded patient data, the patient information system, the imaging devices, and imaging data. For this reason, we selected one control case diagnosed within the same year (+/−7 months) for each malignant tumor. In addition, imaging protocols have evolved over time, with MRI imaging becoming more commonly utilized today than 20 years ago.

Finally, deep learning approaches are a growing tool for radiologic and histologic diagnostics. Deep learning may help in distinguishing benign and malignant signs of rare tumors, like SNIP, as it can overcome subjective interpretative errors. Liu and coworkers explored a deep learning approach in MRI that can distinguish between SNIP and SNIP-SCC [32]. Qi M and coworkers developed a nomogram that combines tumor morphology and ADC values in MRI to facilitate the pre-operative prediction of the malignant transformation of SNIP [33]. Yui and coworkers were able to develop an AI-based diagnostic system for the preoperative prediction of SNIP using nasal video endoscopy [34]. However, a large amount of data and more studies are needed to train the deep learning models managing rare tumors like SNIP.

## 5. Conclusions

Our findings from the pre-operative CT images indicate that bony destruction, remodeling, and invasion of adjacent structures may be signs of SNIP-SCC. While MRI is also recommended as a pre-operative imaging method, MRI safety risks must be considered individually. Given that SNIP is a rare and unpredictable tumor, distinguishing between SNIP and SNIP-SCC poses a diagnostic challenge from the radiological, clinical, and pathological point of view. It is essential to conduct all available pre-operative investigations and not rely solely on one method when planning treatment.

## Figures and Tables

**Table 1:** Clinical characteristics of eight patients with benign sinonasal inverted papilloma (SNIP) and eight with SNIP-associated squamous cell carcinoma (SNIP-SCC).

Clinical Characteristics	SNIP-SCC *n* (8)	SNIP *n* (8)
Male	88% (7)	75% (6)
Female	12.5% (1)	25% (2)
Recurrence	12.5% (1)	62.5% (5)
* SCC synchronous	75% (6)	0%
* SCC metachronous	25% (2)	0%
Mean age at IP diagnosis (years, mean ± SD) *	64 ± 15	50 ± 8
Mean follow-up months (mean ± SD) *	35 ± 18	66 ± 32

* SD = standard deviation; SCC = squamous cell carcinoma.

**Table 2:** CT imaging characteristics of eight patients with benign sinonasal inverted papilloma (SNIP) and eight with SNIP-associated squamous cell carcinoma (SNIP-SCC).

Findings Only Found in SNIP-SCC	SNIP-SCC *n* (8)	SNIP *n* (8)
Orbit invasion	12.5% (1)	0%
Intracranial invasion	25% (2)	0%
Bone defect		
Nasal cavity wall	50% (4)	0%
Maxillary	50% (4)	0%
Ethmoid	25% (2)	0%
Sphenoid	12.5% (1)	0%
Frontal	25% (2)	0%
**Some differences between groups**	**SNIP-SCC *n* (8)**	**SNIP *n* (8)**
Shape of tumor		
Polypoid	50% (4)	75% (6)
Unsharp	50% (4)	25% (2)
Tumor location		
Unilateral	50% (4)	87.5% (7)
Bilateral	50% (4)	12.5% (1)
Nasal cavity wall	100% (8)	100% (8)
Maxillary	50% (4)	62.5% (5)
Ethmoid	87.5% (7)	75% (6)
Sphenoid	25% (2)	0%
Frontal	25% (2)	37.5% (3)
CT enhancement *	87.5% (7/8)	62.5% (5/8) *
Site of hyperostosis on CT		
Nasal cavity wall	0%	25% (2)
Maxillary	12.5% (1)	0%
Ethmoid	0%	25% (2)
Sphenoid	0%	0%
Frontal	12.5% (1)	0%
**No differences between groups**	**SNIP-SCC *n* (8)**	**SNIP *n* (8)**
Mean diameter of tumor, mm (range)	57 (30–87)	48 (20–70)
Surface of the tumor		
Smooth	25% (2)	12.5% (1)
Rough	75% (6)	75% (6)
Tumor calcification	25% (2)	25% (2)

* Contrast-enhanced examination was performed in seven of the eight SNIP-SCC cases and in five of the eight SNIP cases.

**Table 3:** Histologic characteristics of five patients with benign sinonasal inverted papilloma (SNIP) and five with SNIP-associated squamous cell carcinoma (SNIP-SCC).

Only in SNIP-SCC Group	SNIP-SCC (*n* 5)	SNIP (*n* 5)
Nuclear atypia	40% (2)	0%
Maturation disturbance	40% (2)	0%
Dyskeratosis	20% (1)	0%
**More often in SNIP-SCC group**	**SNIP-SCC (*n* 5)**	**SNIP (*n* 5)**
Basaloid hyperplasia	100% (5)	40% (2)
Mitosis in basal cell layer	80% (4)	40% (2)
Loss of epithelial stratification	80% (4)	40% (2)
Mitosis above basal cell layer	40% (2)	20% (1)
Goblet cells	40% (2)	20% (1)
**More often in SNIP group**	**SNIP-SCC (*n* 5)**	**SNIP (*n* 5)**
Clear cell cytoplasm	20% (1)	60% (3)
Respiratory epithelium	20% (1)	40% (2)
Squamous cell metaplasia	40% (2)	60% (3)
Neutrophils	60% (3)	80% (4)
**The same frequencies in both groups**	**SNIP-SCC (*n* 5)**	**SNIP (*n* 5)**
Polarity disturbance	80% (4)	80% (4)
Micro-abscess	60% (3)	60% (3)
Eosinophils	40% (2)	40% (2)
Apoptotic cells	20% (1)	20% (1)
**No observations**	**SNIP-SCC (*n* 5)**	**SNIP (*n* 5)**
Atypical keratinization	0%	0%
Apoptotic keratinocyte	0%	0%
Atypical mitosis	0%	0%

## Data Availability

Patient data is unavailable due to privacy and ethical restrictions.

## Abbreviations

The following abbreviations are used in this manuscript:

ADC	apparent diffusion coefficient
AI	artificial intelligence
CCP	convoluted cerebriform pattern
CT	computed tomography
DCE	dynamic contrast-enhanced
FDG	fluorodeoxyglucose
MRI	magnetic resonance imaging
PET	positron emission tomography
SAR	specific absorption rate
SCC	squamous cell carcinoma
SNIP	sinonasal inverted papilloma
TIC	time intensity curve
